# Study Protocol of a School-Based Randomized Controlled Trial to Promote Cycling to School Among Students in Germany Using Intervention Mapping: The ACTS Project

**DOI:** 10.3389/fpubh.2021.661119

**Published:** 2021-08-09

**Authors:** Dorothea M. I. Schönbach, Palma Chillón, Adilson Marques, Miguel Peralta, Yolanda Demetriou

**Affiliations:** ^1^Department of Sport and Health Sciences, Technical University of Munich, Munich, Germany; ^2^PROFITH “PROmoting FITness and Health Through Physical Activity” Research Group, Sport and Health University Research Institute (iMUDS), Department of Physical Education and Sports, Faculty of Sport Sciences, University of Granada, Granada, Spain; ^3^CIPER, Faculty of Human Kinetics, University of Lisbon, Lisbon, Portugal

**Keywords:** bicycle, active travel to school, program, children, adolescents, intervention mapping

## Abstract

**Background:** Despite a high rate of bicycle ownership, the prevalence of cycling to school among children and adolescents in Germany has been constantly low. Cycling to school can contribute to meeting the physical activity recommendations, which the majority of children and adolescents in Germany do not meet.

**Methods:** By using intervention mapping, this study protocol describes the systematic planning process of a school-based intervention in Germany aimed to increase the number of days on which students cycle to school and to increase their physical activity levels. To make sure that the intervention will match the needs of students, we conducted a concept mapping study investigating what students need to cycle to school, as perceived by students, parents, and teachers. The logic model of change was based on an integration of the self–determination theory and the social–ecological model. We structured our intervention as two phases, a preparatory phase with weekly components for and a practical phase with a daily repeated component of the targeted behavior. In the 8-week preparatory phase, teachers, parents, and peers will be involved. The content of the 12-week practical phase will involve peers only and was considered promising based on the findings from a systematic review that we conducted to identify the effective strategies of school-based interventions to promote cycling to school among children and adolescents. Overall, our intervention includes 27 behavior change techniques. A researcher, student assistants, teachers, and other collaborators will implement the intervention; a whole-of-school approach with components performed before, during, and after school was chosen. As a study design, we decided to draft a two-arm three-level cluster randomized controlled trial. Both the effect and process evaluation were prepared. In the first instance, approximately 250 students of 12–15 years of age from grade 7 or 8, who attend a secondary school of intermediate or high educational level located in (sub)urban regions in Southern Germany, will pilot the intervention.

**Discussion:** We expect to provide an effective and sustainable intervention for students, which gives insights into the mechanisms of change concerning the behavior of cycling to school and its influence on physical activity levels.

## Introduction

In Germany, up to 98% of children and adolescents until 17 years own a bicycle (1). However, cycling is the rarest mode used by girls and boys overall for commuting to school (2). Additionally, the prevalence of cycling to school in the years from 2003 to 2017 was constantly lower in girls (20.6 vs. 21.5%) compared with boys (23.8 vs. 25.2%) aged 11–17 years (2, 3). Living in a small town (5,000–19,999 inhabitants) and a city (>100,000 inhabitants) or attending an intermediate educational level providing a general education school leaving certificate lowered the chance of cycling to school among children and adolescents in Germany compared with those living in a medium-sized town (20,000–99,999 inhabitants) or attending a high educational level providing a general higher education entrance qualification (3, 4). These associations may vary based on the context (i.e., sampled regions of residential or school area) (5).

In Germany, only 26% of the children and adolescents (girls: 22.4%, boys: 29.4%) aged up to 17 years achieve the physical activity (PA) recommendations proposed by the World Health Organization (6). As the stability of PA patterns among girls and boys is lower in transitional phases (e.g., from childhood to adolescence) (7), the PA prevalence declines with age in Germany (6), which makes it important to counteract this negative trend in this phase of life. According to previous research from England, it is noteworthy that 36% of children and adolescents aged 5–15 years who cycle to school meet the weekly PA recommendations (8). In comparison, only 25% of walkers to school and 22% of neither cyclists nor walkers to school meet these recommendations. Following this, the promotion of cycling to school could be a promising strategy to increase PA levels among children and adolescents.

However, interventions in this research field are not well established (9) and especially interventions implemented in secondary schools (10) involving two grade levels from grade 7 upward (11) are lacking. Previous research recommended the stepwise structured intervention mapping protocol (IM) when planning interventions to change behavior (12). According to this conceptual review, the IM protocol uses theories and evidence, follows a social–ecological approach to intervene at multiple levels, and is characterized by involving the target group and all relevant stakeholders using a participatory approach. The authors concluded that the best possible intervention with the best chance of effectiveness can be expected when following this detailed and systematic protocol.

Therefore, this study protocol used IM to document the systematic planning process of a school-based intervention based on a combination of the social–ecological model and the self–determination theory. It is designed as a two-arm three-level cluster randomized controlled trial (RCT) with a pre- and post-measurement for the effect evaluation before and after the 5-month period of implementation. As the primary aim, the planned intervention should increase the number of cycling days to school and as a secondary aim, should increase the total moderate-to-vigorous physical activity (MVPA) among children and adolescents aged 12–15 years from grade 7 or 8 attending secondary schools of intermediate or high educational levels located in (sub)urban regions (small town, medium-sized town, city) in Southern Germany.

## Methods and Analysis

**Figure 1:**
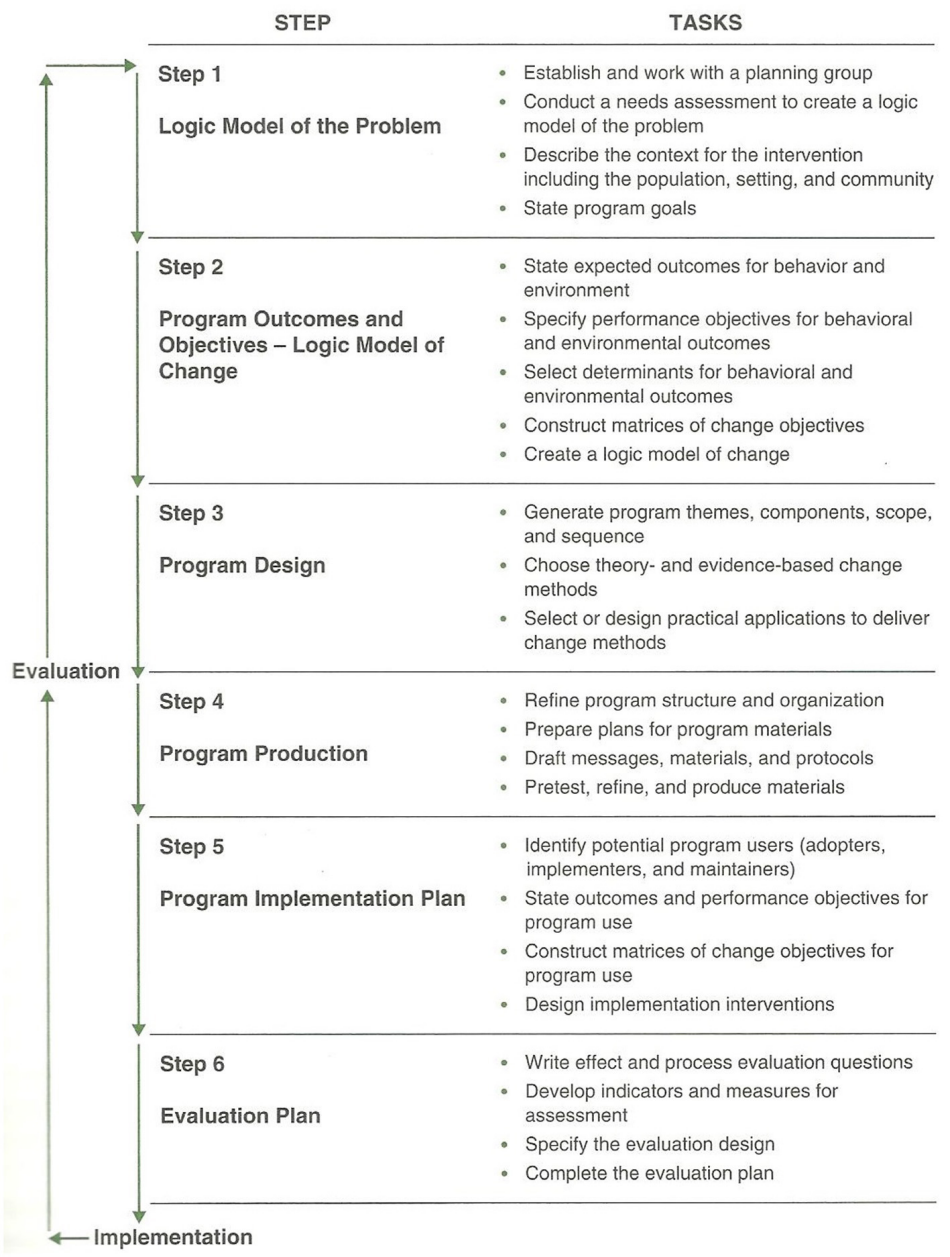
Steps and tasks of the intervention mapping protocol (13) (p. 13).

In this study protocol, IM was used. IM describes an iterative process, which consists of six steps (i.e., logic model of the problem, logic model of change, program design, program production, program implementation plan, and evaluation plan) divided into several tasks described in Figure 1 (13).

This study protocol includes the following terms, which explain the most crucial tasks allocated to steps one to three of the IM protocol. In step one, we defined the needs assessment as “the collection and analysis of information that relates to the needs” (14) (p. 314) of our identified high-risk population of cyclists to school, which help determine the facilitators and barriers of their behavior. For the construction of the matrix in step two, we used the following definitions of “performance objectives” and “change objectives.” Performance objectives are observable and specific behaviors, which are judged necessary to meet the desired aims of our intervention (i.e., who needs to do what) (13) and were allocated to modules (A, B, C, etc.) in the study matrix. The combination of determinants for behavioral outcomes and performance objectives lead to change objectives (13). In step three, intervention components were defined as different packages of contents (15), which were allocated to the different modules in order to address the changes needed according to the identified performance objectives. The composition of components will define the success of our intervention due to their direct relatedness to change methods (16). A change method (also technique) “provides evidence for how change may occur” (13) (p. 17).

### Logic Model of the Problem

In 2019, the ACTS project was initiated. It was aimed at promoting active commuting to school (ACTS) with a particular focus on cycling to school in Europe. To plan interventions is part of this project. This project involves six research institutes from Poland, Czech Republic, Portugal, the Netherlands, and Germany, which were set as the planning group. For each country, an intervention adapted to the needs of the local context is planned. The intervention described here aims to address students aged 12–15 years attending grade 7 or 8 at secondary schools of intermediate or high educational levels located in sub(urban) regions (small town, medium-sized town, city) in Southern Germany. We involved teachers in the planning process of the intervention to ensure that the implementation will be feasible in their community, at their school, and with their students. Therefore, we sent teachers the draft of our planned intervention and asked for their feedback, which we considered in this study protocol.

**Figure 2:**
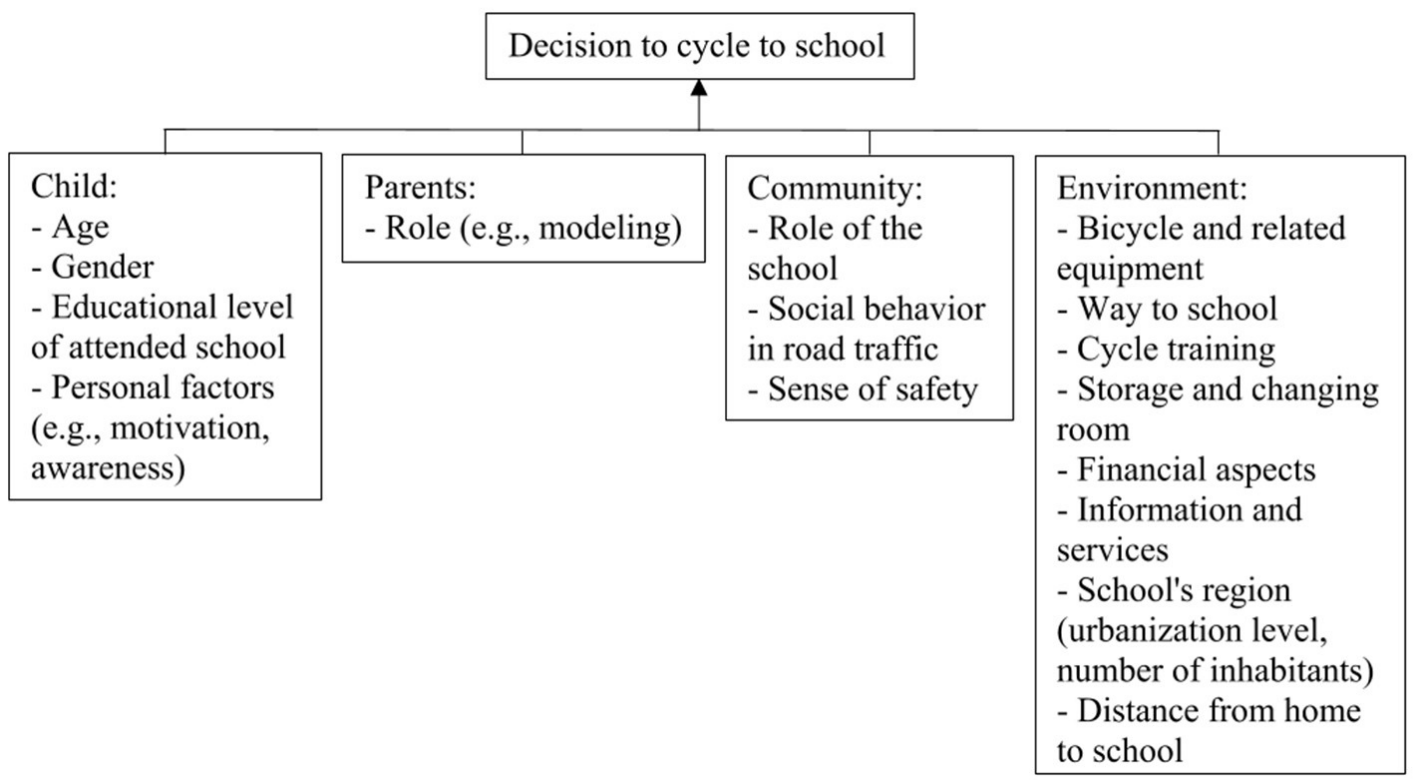
Logic model of the problem: Factors that influence cycling to school based on concept mapping (5, 17).

In step one of the IM protocol, the logic model of the problem was created (see Figure 2). Here, the needs of students to cycle to school daily were assessed using a concept mapping study (17). In total, 136 students aged 12–15 years attending grade 7 or 8 at three different secondary schools of different educational levels located in different sub(urban) regions in Southern Germany participated in the study. For a more comprehensive understanding of the behavior of the students, concept mapping was also performed among students' parents (*n* = 58) and teachers (*n* = 29) of both genders female and male, whereby the low retention rate of fathers did not permit a separate gender analysis. As needs to cycle to school daily, a “bicycle and related equipment,” the “way to school,” and “personal factors” were mentioned by all three analyzed samples of students, mothers, and teachers. Additionally, students mentioned “cycle training,” mothers mentioned the “role of the school,” and the teachers mentioned “storage and changing room,” “financial aspects,” as well as “information and services.” Furthermore, “social behavior in road traffic” was mentioned by girls only, “role of parents” by mothers and female teachers, and “sense of safety” by female teachers. As none of these mentioned needs stood out as particularly (un)important and/or (un)feasible, we treated all of them as equally relevant. In this study, almost all students owned a bicycle (girls: 87.8%, boys: 100%) and all were able to cycle (5). When examining the habits of the students in cycling to school, we found that approximately two-thirds of students stated to sometimes cycle to school (girls: 44.4%, boys: 72.9%), of whom approximately one-third cycled to school daily (girls: 31.6%, boys: 37.3%). On average, students generally cycled to school on 2.3 ± 2.0 days per week (girls: 1.6 ± 2.0, boys: 2.7 ± 2.0). Moreover, the following correlates were identified to be negatively associated with cycling to school habits of students: (a) being a girl, (b) increasing age (mainly in girls), (c) attending an intermediate educational level in combination with a suburban region of the school, (d) attending a school located in a small town (mainly in girls) or a medium-sized town, (e) living further away from school, and (f) having parents not using a bicycle to commute to work.

The primary aim of the planned intervention is to increase the number of days on which students cycle to school. As a secondary aim, the planned intervention should increase the total MVPA in students due to positive changes in their cycling to school behavior.

### Logic Model of Change

According to the previous IM step, complementary and stimulating impulses of behavioral (including personal factors) and situational (including social and physical environment) approaches are relevant for the successful promotion of cycling to school. All circumstances of the external reality of the students embody the environment (18). We defined the term “social environment” as all political–social–cultural factors (including parents, teachers, peers, school and its policies, and social norms), in which the student lives in, is educated, and interacts (19, 20). The term “physical environment” includes all factors related to the structural conditions, such as the (functional) quality of equipment, financial aspects, transportation system, design of the infrastructure, services, and the distance from home to school (20–22).

**Figure 3:**
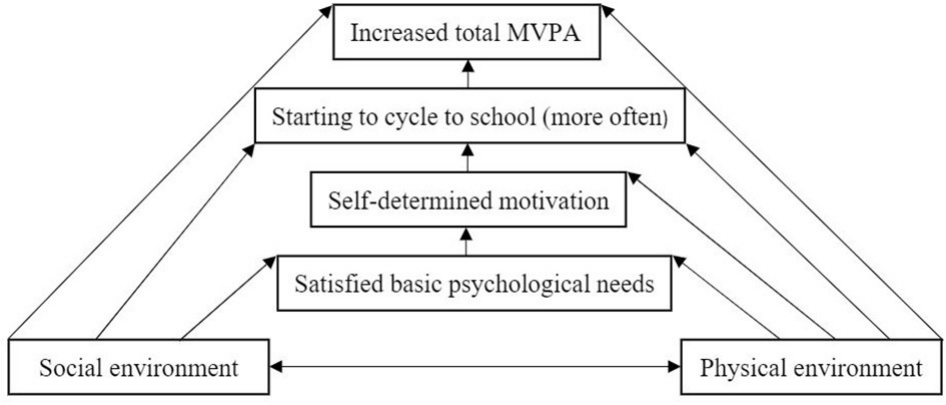
Logic model of change [modified according to (21) (p. 67)]. MVPA, moderate-to-vigorous physical activity.

In step two of the IM protocol, a theoretical model has to be chosen to create the logic model of change. Therefore, two theoretical models were integrated as already described in a previous study protocol (23). This integration illustrates why the situational approach influences the behavior of the students and how the behavioral and situational approaches interrelate and interact (21) (see Figure 3): (a) The social–ecological model of the correlates of active transportation shows “the complex interaction of multiple levels of factors [i.e., individual, interpersonal, community, built environment, policy (19)] affecting decisions to be active” (21) (p. 57). This theoretical model was chosen as we identified multiple levels in our concept mapping study at which we need to intervene (i.e., individual, interpersonal, community, environment) (see Figure 2). (b) A sub-theory of the self–determination theory, the basic psychological needs (BPNs), emphasizes that the support and satisfaction of autonomy, competence, and relatedness lead to a more self-determined form of motivation toward a specific behavior (21, 24). This theoretical model was chosen as motivation (personal factors) of students was identified in our concept mapping study to play a role in their decision to cycle to school (see Figure 2).

Based on the social and physical environment, BPNs can be either satisfied or frustrated leading to a certain degree of motivation, which is crucial for the decision process of the students to cycle to school and the influence on total MVPA. Following this logic model of change, Table 1 illustrates the matrix of performance objectives and determinants targeting the promotion of cycling to school.

**Table 1:** Matrix of performance objectives and determinants targeting the promotion of cycling to school.

**Modules**	**Performance objectives**	**Psychological determinants**		
		**Autonomy**	**Competence**	**Relatedness**
A	Researcher communicates to parents and students the purpose and benefits of cycling to school, useful bicycle-related equipment, and feasible mixed methods when living too far away from school.	Students can choose between different options.	Students are aware of the purpose/benefits and solutions to tolerate adverse conditions (e.g., bad weather, heavy schoolbags) when cycling to school.	Students perceive social support.
B	Researcher seeks help from parents and teachers (e.g., parents do not drive their child to school by car, parents/teachers motivate their child/students to cycle to school, parents/teachers are role models for their child/students by cycling to work regardless of the weather condition and wearing a helmet, teachers develop a cycling-to-school-mission-statement).	Students are personally responsible and extensively independent in planning how to get to school.	Students feel empowered to cycle to school when encouraged.	Students perceive a new social norm, social support from parents and teachers, and social cohesion. Students learn how to establish a cycling routine regardless of the weather condition and wear a helmet through the role modeling of parents and teachers.
C	Researcher shows helmet-compatible hairstyle to students and parents.	Students make their own decisions, which helmet-compatible hairstyle they want to do.	Students know, which hairstyles are helmet-compatible, and can do them.	Students establish the new social norm to wear a helmet and receive social support from parents who can help them to do their helmet-compatible hairstyle. Peers serve as role models.
D	Students and parents plan routes and stops so that students can cycle to school together.	Students are free in choosing the best route to cycle to school.	Students feel proud to cycle to school on their own chosen routes.	Students feel involved in the planning process, interact socially with peers and parents, establish a new social norm at school, and perceive social support from peers and parents. Peers serve as role models.
E	Students, parents, and teachers plan cycling-to-school-events.	Students have the freedom to choose what kind of events they want to plan.	Students are proud of the successful realization of their planned events.	Students feel involved in the planning process of the intervention, perceive social support from peers, parents, and teachers, and interact with peers, parents, and teachers. Peers serve as role models.
F	Students set goals on how often they want to try to cycle to school per week.	Students decide on their own how often they want to try to cycle to school per week.	Students successfully reach their set goals.	Students establish a social norm and perceive social support from peers. Peers serve as role models.
G	Researcher ensures that bicycles of students are roadworthy and provides required bicycle-related equipment if necessary.	Students have the chance to engage in cycling to school if they want to.	Students trust in the safety of their bicycles.	Students perceive the principle of equal opportunities and social support.
H	Students personalize bicycle-related equipment.	Students decide on their own how to make their bicycle-related equipment more attractive to themselves.	Students receive positive feedback for their art from the teacher and peers, which encourage them to present it on-road.	Students identify with the intervention and their bicycle-related equipment. Students develop group cohesion through personalized bicycle-related equipment as a common identifying feature of participating in the intervention.
I	Students can cycle to school in road traffic (e.g., improve basic cycling skills, know traffic rules, practice social behavior, take part in a final exam).	Students make their own decisions on how to appropriately behave in road traffic.	Students feel safe in applying traffic rules, have confidence in and do not overestimate their cycling skills, and make positive experiences when cycling to school.	Students interact socially with other traffic participants.

### Program Design and Production

As illustrated in Table 2, the intervention will be structured in two phases: (a) preparation for and (b) practice of the targeted behavior. All chosen components and some of their descriptions were based on the findings and conclusions from our concept mapping study (5, 17) and our systematic review (11), which was conducted to identify effective strategies of school-based interventions to promote ACTS by bicycle among children and adolescents. We also used the following documents to design the mobility and traffic education components in the preparatory phase: (a) The content of the three-cycle training sessions off-road was based on a German research report on road safety education concepts for children and adolescents in secondary schools (25). (b) For the session in which theoretical knowledge about traffic rules will be transferred, two guidebooks published by the German Automobile Club (ADAC) (26, 27) were the basis. (c) The content of the cycle training session on-road to practice social behavior in road traffic was also based on these two guidebooks (26, 27). (d) To finally certify the basic cycling skills of students in a final exam, another guidebook of the ADAC was the basis (28). All students will obtain a certificate regardless of their scoring to allow for self-monitoring of their basic cycling skills.

**Table 2:** Program design.

**Phases**	**Modules**	**Components**	**Descriptions**	**Materials**	**Behavior change techniques (11, 29)**
Preparation	A-F	Joint parents', teachers', and students' evening	- General information for students, parents, and teachers: ➜ Purpose and benefits of cycling to school (e.g., health, emotion, environment) ➜ Options when living too far away from school (e.g., splitting the way to school into active and passive parts) ➜ The role of parents and teachers (e.g., role modeling, motivators) ➜ Useful bicycle-related equipment (e.g., clothes, carrier systems) ➜ Helmet-compatible hairstyles - Parents help students to develop a cycling-to-school-plan by letting students tell peers where they live and forming small groups when living close together to determine a joint route and stops - Teachers develop a cycling-to-school-mission-statement as part of a new school policy - Students, parents, and teachers determine three cycling-to-school-events - Students set goals in written form	To perform: Computer, projector, paper, pencils, roadmaps To provide students, parents, and teachers with: Online video and booklet of the live meeting	Involving parents and teachers, pros and cons, information about health, emotional, social, and environmental consequences, avoidance/reducing exposure to cues for the behavior, restructuring the physical and social environment, social support (unspecified, practical, emotional), demonstration of the behavior, knowledge transfer, adding objects to the environment, information about antecedents, goal setting (behavior), action planning
	G-H	Bicycle inspection in the presence of parents and provision of required bicycle-related equipment; personalization of bicycle-related equipment		To perform: Tool kits, paper and pencils to document required bicycle-related equipment, paint, paintbrush	Information about antecedents, restructuring the physical environment, social support (practical), adding objects to the environment, involving parents
	I	Three cycle training sessions off-road (improvement of basic cycling skills) (25)	1. Session: Ascending/descending, slow driving, braking, driving in a narrow lane and over obstacles, orientation 2. Session: Keeping distance, handling and driving over obstacles, slalom, orientation 3. Session: Adaptability, parcours	To perform: 1. Session: Old bicycle tires 2. Session: Little sandbags, pool noodles, self-made seesaw and other obstacles, pylons 3. Session: Old bicycle tires, little sandbags, pool noodles, self-made seesaw and other obstacles, pylons	Instruction on how to perform the behavior, behavioral practice/rehearsal, demonstration of the behavior
		Information about traffic rules (26, 27)	e.g., rights and duties, traffic signs, how to enter traffic, penalties, liability, roadworthiness, how to do an emergency call, blind spot	To provide students with: Booklet	Knowledge transfer, instruction on how to perform the behavior
		One cycle training session on-road (practicing social behavior) (26, 27)	e.g., unhurried driving style (adaptation of speed), keeping distance, how to pass a person/vehicle/bus stop, signaling and looking behind when turning left/right, crossing roads/intersections		Instruction on how to perform the behavior, behavioral practice/rehearsal, demonstration of the behavior, social support (practical), avoidance/reducing exposure to cues for the behavior, feedback on behavior, problem solving, reduce negative emotions, behavior substitution
		Final exam of basic cycling skills in the presence of parents (28)		To perform: Materials from ADAC To provide students with: Certificate	Information about antecedents, behavioral practice/rehearsal, problem solving, reduce negative emotions, feedback on behavior, material reward (behavior), involving parents
Practice	D	Voluntary bicycle train to cycle to school with peers before and after school with an arranged route and stops (incl. three events)		None	Demonstration of the behavior, behavioral practice/rehearsal, behavior substitution, habit formation, habit reversal, adding objects to the environment, social support (unspecified, practical, emotional), restructuring the physical and social environment, information about antecedents, avoidance/reducing exposure to cues for the behavior

*ADAC, German Automobile Club; incl., inclusive*.

Each component could be linked to at least one behavior change technique as proposed in the taxonomy v1 (29) and supplemented by our systematic review (11). Overall, 27 different techniques were applied to the components of our intervention.

### Program Implementation Plan

For convenience, the three secondary schools included in our concept mapping study were asked to pilot the intervention. Two more secondary schools, each characterized by similar features as the already recruited ones, that is, in terms of regions, educational levels, and grades, will be searched by sending random invitation letters. Similar random invitation letters will also be sent to recruit secondary schools in the main study.

The school year in Germany starts in fall and ends in summer, whereby the first term ends in spring. In previous research from Norway, seasonal differences in cycling to school were observed between fall (52%), winter (3%), and spring (51%) (30) as well as between winter (12%) and summer (22%) (31). Also in Germany, cycling to school decreases in winter (32). Therefore, the implementation of the intervention should start in fall and end in spring (i.e., during the first term of the school year). According to the preparatory and practical phases, individual components will be implemented one after another at the participating secondary schools over a period of 5 months (see Table 3). As implemented components will prepare students for the possibly more difficult (weather) conditions in winter, we do not expect a negative seasonal influence on the effectiveness of the intervention. For example, information about appropriate clothes will be provided during the joint parents', teachers', and students' evening. Furthermore, parents and teachers will be reminded of serving as role models so that the 12- to 15-year-olds learn how to establish a cycling routine regardless of (weather) conditions in winter. Students will feel safer and more confident in dealing with difficult road conditions after participating in the cycle training, which will improve their basic cycling skills and practice them on-road (e.g., handling obstacles and appropriate driving style). The bicycle train will increase the motivation and safety of students through positive experiences when accompanied by and interacting socially with peers instead of cycling to school alone in the darkness. Furthermore, the bicycle train will establish a new social norm (i.e., cycling to school as an activity throughout the whole year, including winter and not only in summer). The intervention will follow a whole-of-school approach as we designed components that will take place before, during (i.e., in art and physical education (PE) lesson), and after school. At each participating secondary school, a person of contact will be defined who will act as a coordinator for implementing the intervention. Furthermore, the person of contact will interact with the responsible implementers at their school (i.e., PE and art teacher) and a researcher as well as the project manager at the Technical University of Munich (TUM) who can be called or e-mailed any time in case of occurring questions or problems. The researcher from TUM will be in touch with the person of contact to organize data collections and the implementation of components, which will be led by the researcher and student assistants from TUM. As our aim was to draft a sustainable intervention, we will provide secondary schools with all materials needed for the replication of the intervention and initiate several collaborations with government facilities (i.e., police) and nongovernmental organizations (e.g., ADAC) free of charge that can be continued after we will have left schools.

**Table 3:** Program implementation plan.

**Phases**	**Weeks**	**Components**	**Locations**	**Time frame**	**Implementers**	**Tasks to be prepared**
Preparation	1	Joint parents', teachers', and students' evening	School assembly hall and online	After regular school hours (1 × ca. 150 min)	Person of contact at school	1. Finding a possible date and communication to the researcher at TUM2. Handing invitations out to students for their parents and to teachers3. Preparing assembly hall (e.g., chairs, media)
					Researcher and student assistant from TUM	1. Drafting an invitation letter for parents and teachers2. Preparing lecture/materials (i.e., booklet, video)3. Preparing assembly hall (e.g., chairs, media)
	2	Bicycle inspection in the presence of parents and provision of required bicycle-related equipment	School's playground	After regular school hours (1 × ca. 180 min)	Person of contact at school	1. Finding a possible date and communication to the researcher at TUM2. Handing invitations out to students for their parents3. Helping to prepare the event4. Handing bicycle equipment out to students
					Researcher and student assistant from TUM	1. Contacting the ADFC, asking for a collaboration, and what is needed to perform the event2. Drafting an invitation letter for parents3. Preparing the event (e.g., materials)4. Organizing missing bicycle equipment
					ADFC	Preparing and performing bicycle inspection
	2	Personalization of bicycle-related equipment	Classroom	During regular art lesson (1 × 45 min)	Art teacher	Supervision
	3–7	Cycle training sessions (incl. improvement of basic cycling skills, information about traffic rules, practicing social behavior)	Off-/on-road	Once per week during regular PE lesson (90 min): Off-road (3x), knowledge transfer (1x), on-road (1x)	PE teacher	Realization of lesson plans
					Researcher from TUM	1. Drafting detailed lesson plans2. Preparing materials (i.e., obstacles, booklet)3. Handing lesson plans and materials out to teachers
	8	Final exam of basic cycling skills in the presence of parents (incl. provision of a certificate)	School's playground	After regular school hours (1x ca. 180 min)	Person of contact at school	1. Finding a possible date and communication to the researcher at TUM2. Handing invitations out to students for their parents3. Helping to prepare the event
					Researcher and student assistant from TUM	1. Contacting the ADAC, asking for a collaboration, and what is needed to perform the event2. Drafting an invitation letter for parents3. Preparing the event (e.g., drafting certificates for each participating student)
					ADAC	Preparing and performing final exam
Practice	9–20	Voluntary bicycle train to cycle to school among peers with an arranged route and stops (incl. events)	On the way to/from school	5x to and 5x from school per week (i.e., before and after school) with 1 event per month	Researcher from TUM	Contacting the responsible police station, asking for a collaboration, and what is needed to perform the event
					Police	Supporting the cycling-to-school-events (e.g., kick-off event)

*ADAC, German Automobile Club; ADFC, German Cyclist's Club; ca., circa; incl., inclusive; PE, physical education; TUM, Technical University of Munich*.

### Evaluation Plan

To report the findings of our planned evaluation, the “CONSORT 2010 statement: extension to cluster randomized trials” (33) will be followed.

#### Study Design

**Figure 4:**
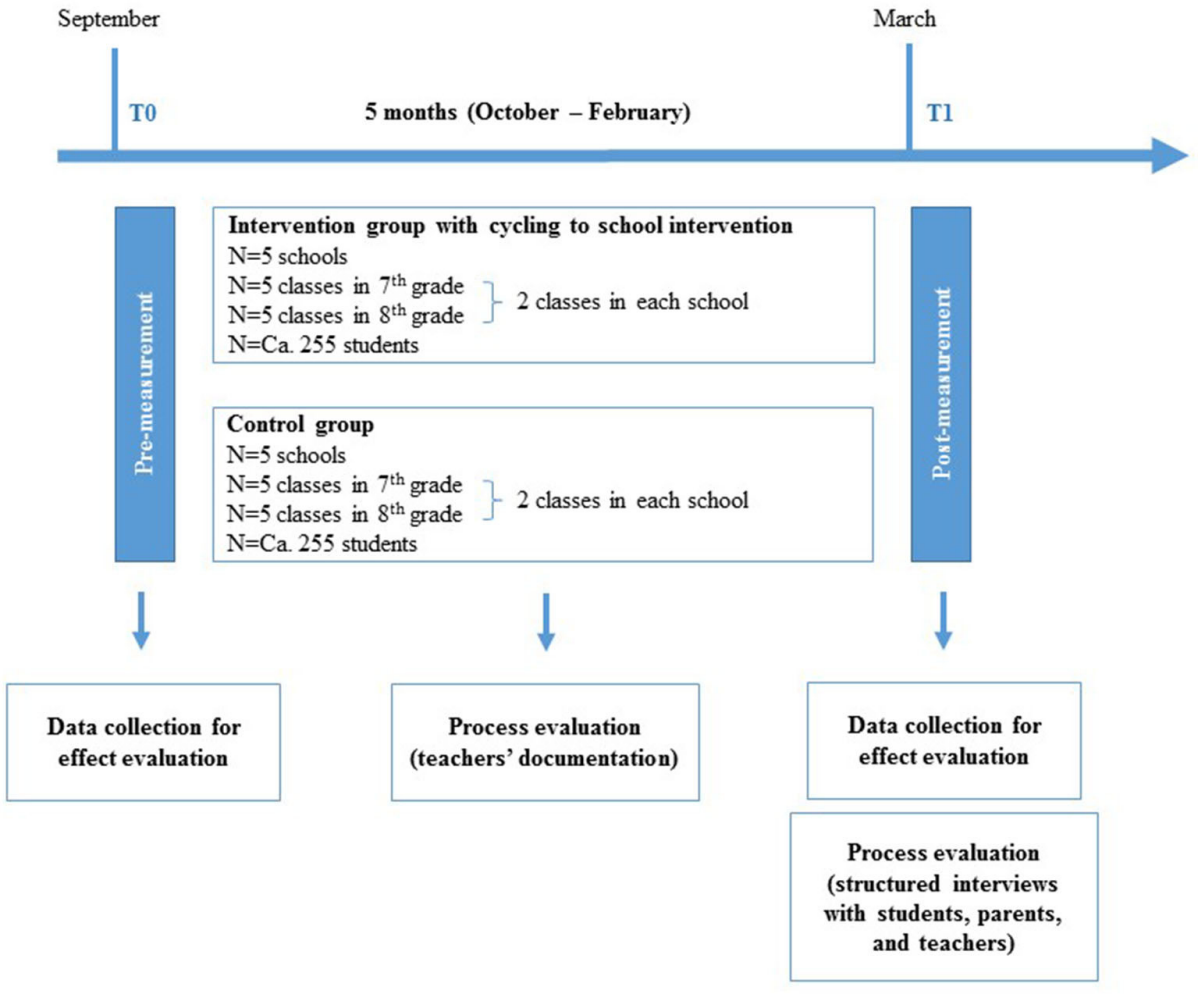
Study design of the planned cycling to school intervention. *N*, sample size.

The main study is planned as a two-arm (i.e., intervention and control group (IG; CG)) three-level cluster (i.e., students in classrooms in schools) RCT (see Figure 4), whereby a simple randomization technique (i.e., flipping a coin) on school-level was chosen. For convenience, the pilot study will follow a quasi-experimental study design as a non-RCT. Directly before and after the implementation of the intervention, a pre- and post-measurement will take place as part of the effect evaluation. Furthermore, the process evaluation will take place during and after the implementation of the intervention. Based on the intraclass correlation coefficient (ICC) of 0.2 calculated for the variance of days per week students cycled to school between the three secondary schools in our concept mapping study (17), we assigned more than one secondary school to the IG and CG. No treatment will be delivered to the CG. In the main study, approximately 255 students belonging to five schools with two classes in grade(s) 7 and/or 8 will be in the IG and CG, respectively. However, approximately 150 students belonging to three schools will be in the IG and 100 students belonging to two schools in the CG when piloting the intervention.

#### Sample Size Determination

The optimal sample size for our chosen study design in the main study was calculated based on a formula by Rutterford et al. (34). This formula considers the confidence level (97.5%), power (80%), variance of days per week in cycling to school at the individual level [4.1 days (5)], our estimated clinically important difference in treatment means of days per week in cycling to school at the individual level (1.75 days), number of students per secondary school (based on the mean value in our concept mapping study: 51 students), and ICC of days per week students cycled to school at school level [0.2 days (17)]. According to this formula, the required number of students per intervention condition is 231. For the planned pilot study in the first instance, 10% of the main study's calculated sample size is recommended (35, 36) (i.e., 23 students per arm).

#### Measuring Instruments

To perform the effect evaluation of the planned intervention, several measuring instruments were chosen (see Table 4). Furthermore, the content of the process evaluation was defined.

**Table 4:** Measuring instruments for the effect evaluation.

**Outcomes**	**Variables**	**Instruments**	**Descriptions**
Primary outcome	Mode, frequency, and duration of ACTS	Two valid self-report questions (37)	Behavior of cycling to school will be measured retrospectively for 5 weekdays: (a) Thinking about the last school week, how did you get to school/home from school each day?. Possible answers are walking, cycling, car, motorcycle, bus, underground/train/tram, or other. (b) Write beside the mode the journey start and end time.
Secondary outcome	Total MVPA in min per day	Accelerometers (ActiGraph wGT3X-BT)	On 7 consecutive days (38, 39), thigh-mounted (40) accelerometers with a sample rate of 30 Hz should be worn from waking up until going to bed except during water activities for a minimum of 8 h on a minimum of 3 weekdays and 1 weekend day (38, 39). Collected data will be downloaded using an epoch length of 1 s (38, 39). For wear time validation, the algorithm from Choi et al. will be applied (41). For data analysis, cut points from Hänggi et al. will be applied (42). For initialization and data processing of accelerometers, ActiLife will be used.
Moderators	Sociodemographic characteristics (5, 20)	Thirteen self-report questions	(a) Age/grade (b) Gender (c) Bicycle ownership/roadworthiness (d) Ability to cycle (e) Shortest distance from home to school by bicycle using Google Maps (43) (f) Zip code of the school (educational level, region) (g) Subjective socioeconomic status using the reliable “MacArthur Scale of subjective social status—youth version” (44) (h) Parents' restriction/allowance in terms of letting their child cycle to school (i) Family car ownership (45)
	Daily weather conditions (46)	Meteo Info	(a) Average rainfall in l/m^2^ (b) Average wind speed in km/h (c) Relative humidity in % (d) Temperature in °C (average, minimum, maximum)
Mediators	Regulatory styles of motivation types (amotivation, extrinsic motivation, intrinsic motivation)	“German behavioral regulation in cycling to and from school” (BR-CS) as self-report questionnaire based on the valid and reliable “Behavioral regulation in active commuting to and from school” (BR-ACS) questionnaire (47)	Twenty-three items with three or four items per regulatory style will be rated on a five-point Likert scale ranging from strongly disagree, disagree, neutral, agree to strongly agree.
	Satisfaction of the three BPNs autonomy, competence, and relatedness	“German basic psychological needs satisfaction in cycling to and from school scale” (BPNS-CS) as self-report questionnaire based on the valid and reliable “Basic psychological need satisfaction in active commuting to and from school scale” (BPNS-ACS) (48)	Twelve items with four items per need will be rated on a five-point Likert scale ranging from strongly disagree, disagree, neutral, agree to strongly agree.
	Cycling skills	Reliable practical cycling skills exam off-road (49)	Seven basic tasks representing essential situations in road traffic will be examined (i.e., slalom, slow driving, ascending/descending, driving in a narrow lane, turning left, driving an eight with one hand, braking between two lines).

*ACTS, active commuting to school; BPNs, basic psychological needs; MVPA, moderate-to-vigorous physical activity*.

To perform the pre- and post-measurement for the effect evaluation before and after the implementation of the intervention, participating classes will be divided into two small groups by the researcher and student assistants from TUM during two regular consecutive PE lessons with a total duration of 90 min. One group will answer the self-report questions in paper/pencil questionnaires, while the other group will perform a practical cycling skills exam off-road. Students who completed the questionnaire will be sent to the other group to complete the cycling skills exam and the other way around. At the end of the two regular consecutive PE lessons, accelerometers will be handed out and their handling will be explained to students (see Table 4). While wearing the accelerometers, weather conditions will be documented daily by a student assistant from TUM.

For the process evaluation during the implementation of the intervention, each art and PE teacher from each class that received the intervention will be asked to document in written form the dosage of their own intervention delivery (i.e., was the content of components implemented correctly), fidelity (i.e., what content of components was not implemented correctly and why), and any adaptions (i.e., what changes were made to the content of components during implementation). Thus, the dimension of the implementation of the intervention can be determined. Additionally, four willing students (two girls and two boys), four parents (two mothers and two fathers), and each art and PE teacher from each class that received the intervention will be asked in a structured online interview individually scheduled after the implementation of the intervention how they perceived the organization and content of the delivered intervention. They will also be asked to give feedback on their (dis)satisfaction regarding the intervention and to think about how it can be improved.

#### Data Handling, Storage, and Monitoring

Data will be collected using pseudonyms. Therefore, students will be instructed to create a six-digit ID code themselves to connect data throughout data collections and to ensure anonymity. Data collected for the effect evaluation will be entered in *SPSS*. Over a period of at least 10 years, data sets will be stored on central servers of TUM administered by the Leibniz–Rechenzentrum meeting the high standards of data safety in Germany. Only the researcher and manager of the ACTS project at TUM will get access to data sets, which means that anonymous data will not be made available for open access after the end of the study.

#### Statistical Analysis

To analyze the effectiveness of our intervention, a multiple group analysis considering the treatment factor (IG vs. CG) will be performed in a structural equation modeling using *R*. In this analysis, variances in the number of cycling days to school and MVPA due to belonging to different secondary schools will be considered in the following four levels: (a) repeated measurements for each student, (b) students, in which the repeated measurements are nested, (c) the class, to which each student belongs, and (d) the school, in which classes are nested. Subsequently, potential moderators (e.g., sociodemographic characteristics of the students) will be added to the model, and cycling to school will be regressed onto potential mediators (i.e., autonomy, competence, and relatedness) in levels (b–d). Furthermore, gender will be considered as a covariate in the analysis.

## Discussion

This study protocol describes the systematic planning process and design of the 5-month school-based ACTS intervention in Germany aiming to promote cycling to school among 12- to 15-year-olds. It used IM and a combination of the social–ecological model and the self–determination theory.

The decision to publish the detailed process of how our intervention was planned can be seen as a major strength as we demonstrate transparency of our structured procedure for other intervention planners. To the best of our knowledge, this is the first time that IM was used to plan an intervention aimed at the promotion of cycling to school. Our planned intervention has also several strengths: We chose the school setting for our intervention because it is regarded as ideal (50–53). According to the “Standing Conference of the Ministers of Education and Cultural Affairs” in Germany (54), the task to provide mobility and traffic education is assigned to schools, which we support with our planned intervention. Our intervention will contribute to closing the currently existing research gap by focusing on secondary schools where ACTS, especially cycling to school, is currently the least implemented activity (10) and generally, in an early development stage (9) with a lack of evidence for effectiveness in the majority of existing school-based interventions (11). We decided to focus on the promotion of cycling to school in winter (32) among the high-risk ACTS group of 12- to 15-year-olds (55) attending grade 7 or 8 (11) to expand the current state of research. As recommended, we chose a whole-of-school approach (56), a multi-level approach (21, 57), and a combination of objective as well as subjective measuring instruments to accurately assess the PA level during cycling (58). To prevent negative experiences on-road (e.g., accidents), we will first provide some theoretical and practical components off-road. Instead of choosing a top-down approach, we partly chose a participative approach to consider the opinion of the target group (i.e., students) and relevant stakeholders (i.e., parents and teachers), to address the local context appropriately, and to ensure the feasibility of the implementation of the intervention. The success of this approach will be controlled in the process evaluation. Furthermore, we will initiate collaborations (e.g., with the ADAC and police) free of charge and provide all necessary materials so that schools could continue the work beyond the duration of the intervention, which will ensure sustainability. Finally, we determined the appropriate sample size for an adequately powered effect evaluation, will control for potential mediators and moderators in our analysis (57) and will pilot the intervention. The purpose of piloting is to pre-test the effectiveness, acceptance, and feasibility of the intervention, as it will later be performed in the main study, in accordance with the planned organizational procedure and chosen measuring instruments for data collection described in this study protocol but by using a smaller sample size and a weaker, that is, quasi-experimental, study design.

However, the following limitations have to be considered. In general, following the IM protocol was a time-consuming process making it difficult to fulfill each of its sub-steps (59). The time factor is also why the opinion of the target group could not be considered in every step (e.g., how students would design the intervention) but the IM protocol is normally only followed by researchers anyway (60). Besides, one demand mentioned in the needs assessment, that is, storage and changing room, could not be considered in the planned intervention. Moreover, this intervention was designed as an RCT providing a high evidence level (61) but without a follow-up. It will be characterized by a selective sample, that is, recruiting students of intermediate or high educational levels and regional restriction to (sub)urban areas in Southern Germany, and a moderate-term duration so that findings will not be generalizable.

Altogether, using the IM protocol to systematically plan an intervention is a time-consuming and complex procedure for researchers (59, 62, 63) but recommendable as it increases the chance to achieve the defined aim(s) of a planned intervention (12, 63). We suppose that the planned intervention adequately matches the needs of students aged 12–15 years grade 7 or 8 at secondary schools in Southern Germany, covering different educational levels (i.e., intermediate and high) and located in different municipalities urbanized to different levels. Thus, we expect that our effect evaluation will show increasing numbers of days on which students cycle to school and increasing total MVPA. As we considered the opinion from the target group and all relevant stakeholders in the planning process to a certain extent, we expect that the process evaluation will show satisfaction concerning components and the implementation of the intervention as intended.

## Ethics and Dissemination

Before the implementation of the intervention, we will apply for the intervention's approval by the Ethics Commission from TUM and the Bavarian State Ministry for Education and Cultural Affairs. Prior to participating in the intervention, schools, parents, and their 12- to 15-year-old children will have to provide signed consent forms, which will be collected from the person of contact at the participating secondary schools and forwarded to the researcher from TUM.

Any changes made to the methodological procedure described in this study protocol will be reported when publishing the findings of the pilot and main study in international peer-reviewed journals. In addition, the findings will be disseminated through formal presentations at conferences and informal meetings.

## Author Contributions

DS designed the concept of the intervention and drafted the manuscript. YD acquired funding for the project, supervised DS, and commented on the manuscript. PC, AM, and MP commented on the manuscript. All authors read, approved, and agreed to be accountable for the final manuscript.

## Conflict of Interest

The authors declare that the research was conducted in the absence of any commercial or financial relationships that could be construed as a potential conflict of interest.

## Publisher's Note

All claims expressed in this article are solely those of the authors and do not necessarily represent those of their affiliated organizations, or those of the publisher, the editors and the reviewers. Any product that may be evaluated in this article, or claim that may be made by its manufacturer, is not guaranteed or endorsed by the publisher.

## Abbreviations

ACTS: active commuting to school
ADAC: German Automobile Club
BPNs: basic psychological needs
CG: control group
e.g.: for example
etc.: et cetera
ICC: intraclass correlation coefficient
i.e.: that is
IG: intervention group
IM: intervention mapping
min: minutes
MVPA: moderate-to-vigorous physical activity
*n*: sample size
PA: physical activity
PE: physical education
RCT: randomized controlled trial
TUM: Technical University of Munich
vs.: versus.

## References

[1] Federal Ministry of Transport and Digitial Infrastructure (BMVI). Radverkehr in Zahlen. Zahlen, Daten, Fakten. (2014). Available online at: https://www.bmvi.de/SharedDocs/DE/Publikationen/K/radverkehr-in-zahlen.pdf?__blob=publicationFile (accessed Jan 22, 2021).

[2] Reimers AK Marzi I Schmidt SCE Niessner C Oriwol D Worth A. Trends in active commuting to school from 2003 to 2017 among children and adolescents from Germany: the MoMo Study. Eur J Public Health. (2020) 31:2. 10.1093/eurpub/ckaa141 33011779

[3] Reimers AK Jekauc D Peterhans E Wagner MO Woll A. Prevalence and socio-demographic correlates of active commuting to school in a nationwide representative sample of German adolescents. Prev Med. (2013) 56:1. 10.1016/j.ypmed.2012.11.011 23200879

[4] Schöb A. Fahrradnutzung bei Stuttgarter Schülern. Erste ergebnisse einer schülerinnen- und schülerbefragung an stuttgarter schulen 2005. Statistik Informationsmanag. (2006) 11:294–317.

[5] Schönbach DMI Brindley C Reimers AK Marques A Demetriou Y. Socio-demographic correlates of cycling to school among 12-to 15-year olds in Southern Germany. Int J Environ Res Public Health. (2020) 17:24. 10.3390/ijerph17249269 33322403 PMC7763497

[6] Finger JD Varnaccia G Borrmann A Lange C Mensink GBM. Körperliche Aktivität von Kindern und Jugendlichen in Deutschland–Querschnittergebnisse aus KiGGS Welle 2 und Trends. J Health Monit. (2018) 3:1. 10.17886/RKI-GBE-2018-006

[7] Telama R. Tracking of physical activity from childhood to adulthood: a review. Obes Facts. (2009) 2:3. 10.1159/000222244 20054224 PMC6516203

[8] Roth MA Millett CJ Mindell JS. The contribution of active travel (walking and cycling) in children to overall physical activity levels: a national cross sectional study. Prev Med. (2012) 54:2. 10.1016/j.ypmed.2011.12.004 22182478

[9] Yang Y Diez-Roux AV. Using an agent-based model to simulate children's active travel to school. Int J Behav Nutr Phys Act. (2013) 10. 10.1186/1479-5868-10-67 23705953 PMC3668894

[10] Cardon GM Van Acker R Seghers J De Martelaer K Haerens LL De Bourdeaudhuij IMM. Physical activity promotion in schools: which strategies do schools (not) implement and which socioecological factors are associated with implementation?. Health Educ Res. (2012) 27:3. 10.1093/her/cys043 22388742

[11] Schönbach DMI Altenburg TM Marques A Chinapaw MJM. Strategies and effects of school-based interventions to promote active school transportation by bicycle among children and adolescents: a systematic review. Int J Behav Nutr Phys Act. (2020) 17:1 10.1186/s12966-020-01035-1 33183331 PMC7661215

[12] Kok G Peters LWH Ruiter RAC. Planning theory- and evidence-based behavior change interventions: a conceptual review of the intervention mapping protocol. Psicol Reflex Crit. (2017) 30:1. 10.1186/s41155-017-0072-x 32026109 PMC6975763

[13] Bartholomew Eldredge LK Markham CM Ruiter RAC Fernández ME Kok G Parcel GS. Planning Health Promotion Programs. An Intervention Mapping Approach. San Francisco, CA: Jossey-Bass (2016).

[14] World Health Organization (WHO). Needs Assessment. (2021). Available online at: https://www.who.int/health-cluster/resources/publications/hc-guide/HC-Guide-chapter-10.pdf?ua=1 (accessed Jan 22, 2021).

[15] Collins LM Trail JB Kugler KC Baker TB Piper ME Mermelstein RJ. Evaluating individual intervention components: making decisions based on the results of a factorial screening experiment. Transl Behav Med. (2014) 4:3. 10.1007/s13142-013-0239-7 25264464 PMC4167900

[16] Assistant Secretary for Planning and Evaluation (ASPE). Core Intervention Components: Identifying and Operationalizing What Makes Programs Work. What do we Mean by “Core Components”? (2013). Available online at: https://aspe.hhs.gov/report/core-intervention-components-identifying-and-operationalizing-what-makes-programs-work/what-do-we-mean-core-components (accessed Jan 22, 2021).

[17] Schönbach DMI Vondung C Hidding LM Altenburg TM Chinapaw MJM Demetriou Y. Gender influence on students, parents, and teachers' perceptions of what children and adolescents in Germany need to cycle to school: a concept mapping study. Int J Environ Res Public Health. (2020) 17:18. 10.3390/ijerph17186872 PMC7557880 32962261

[18] Hurrelmann K. Jugendliche als produktive realitätsverarbeiter: zur neuausgabe des buches “lebensphase jugend”. Diskurs Kindheits Jugendforschung. (2012) 1:89–100.

[19] Larouche R Ghekiere A. An ecological model of active transportation. In: Larouche R, editor. Children's Active Transportation. Amsterdam: Elsevier. (2018) p. 93–103.

[20] Pont K Ziviani J Wadley D Abbott R. The model of children's active travel (M-CAT): a conceptual framework for examining factors influencing children's active travel. Aust Occup Ther J. (2011) 58:3. 10.1111/j.1440-1630.2010.00865.x 21599678

[21] Zhang T Solmon M. Integrating self-determination theory with the social ecological model to understand students' physical activity behaviors. Int Rev Sport Exerc Psychol. (2013) 6:1. 10.1080/1750984X.2012.723727

[22] Institute of Medicine (IOM). Does the Built Environment Influence Physical Activity?. Examining the Evidence. (2005). Available online at: http://onlinepubs.trb.org/onlinepubs/sr/sr282.pdf (accessed Jan 22, 2021).

[23] Chillón P Gálvez-Fernández P Huertas-Delgado FJ Herrador-Colmenero M Barranco-Ruiz Y Villa-González E. A school-based randomized controlled trial to promote cycling to school in adolescents: the PACO Study. Int J Environ Res Public Health. (2021) 18:4. 10.3390/ijerph18042066 33672550 PMC7923771

[24] Ryan RM Deci EL. Self-determination Theory. Basic Psychological Needs in Motivation, Development, and Wellness. New York, NY: The Guilford Press (2017).

[25] Voll S Moritzer L Gehlert T. Ganzheitliche Verkehrserziehung für Kinder und Jugendliche Teil 5: Konzept Radfahrausbildung (Sekundarstufe I). (2020). Available online at: https://repository.difu.de/jspui/bitstream/difu/578176/2/fb_69_mob_teil_5_web.pdf (accessed Jan 22, 2021).

[26] Allgemeiner Deutscher Automobil-Club e,.V. (ADAC). Fahrradfahren – Aber Richtig!. Regeln, INFORMATIONEN und Tipps. (2018). Available online at: https://www.adac.de/-/media/pdf/rechtsberatung/fahrradfahren.pdf (accessed Jan 22, 2021).

[27] Allgemeiner Deutscher Automobil-Club e,.V. (ADAC). Das Fahrrad-Heft. Tipps und Infos für alle, die Gerne mit Dem Rad Unterwegs Sind. (2018). Available online at: https://www.adac.de/-/media/pdf/vek/verkehrserziehung/das-fahrrad-heft.pdf (accessed Jan 22, 2021).

[28] Allgemeiner Deutscher Automobil-Club e,.V. (ADAC). Jugend-Fahrradturnier. Wer wird Fahrrad-Champion?. Aufgaben, Wertung und Bauanleitung. (2017). Available online at: https://www.adac.de/-/media/pdf/adac-regionalclubs/nordrhein-westfalen/verkehr-und-sicherheit/jugend-fahrradturnier-bauanleitung.pdf (accessed Jan 22, 2021).

[29] Michie S Richardson M Johnston M Abraham C Francis J Hardeman W. The behavior change technique taxonomy (v1) of 93 hierarchically clustered techniques: building an international consensus for the reporting of behavior change interventions. Ann Behav Med. (2013) 46:1. 10.1007/s12160-013-9486-6 23512568

[30] Børrestad LAB Andersen LB Bere E. Seasonal and socio-demographic determinants of school commuting. Prev Med. (2011) 52:2. 10.1016/j.ypmed.2010.12.006 21182855

[31] Fyhri A Hjorthol R. Children's independent mobility to school, friends and leisure activities. J Transp Geogr. (2009) 17:5. 10.1016/j.jtrangeo.2008.10.010

[32] Müller S Mejia-Dorantes L Kersten E. Analysis of active school transportation in hilly urban environments: a case study of Dresden. J Transp Geogr. (2020) 88:102872. 10.1016/j.jtrangeo.2020.102872

[33] Campbell MK Piaggio G Elbourne DR Altman DG. Consort 2010 statement: extension to cluster randomised trials. BMJ. (2012) 345:e5661. 10.1136/bmj.e5661 22951546

[34] Rutterford C Copas A Eldridge S. Methods for sample size determination in cluster randomized trials. Int J Epidemiol. (2015) 44:3. 10.1093/ije/dyv113 26174515 PMC4521133

[35] Treece EW Treece JW. Elements of Research in Nursing. St. Louis, MI: Mosby (1982).

[36] Connelly LM. Pilot studies. Medsurg Nurs. (2008) 17:411–12. 19248407

[37] Chillón P Herrador-Colmenero M Migueles JH Cabanas-Sánchez V Fernández-Santos JR Veiga ÓL. Convergent validation of a questionnaire to assess the mode and frequency of commuting to and from school. Scand J Public Health. (2017) 45:6. 10.1177/1403494817718905 30747037

[38] Bachner J Sturm DJ García-Massó X Molina-García J Demetriou Y. Physical activity-related profiles of female sixth-graders regarding motivational pychosocial variables: a cluster analysis within the CReActivity Project. Front Psychol. (2020) 11:580563. 10.3389/fpsyg.2020.580563 33262728 PMC7686241

[39] Bachner J Sturm DJ Demetriou Y. Accelerometer-measured physical activity and sedentary behavior levels and patterns in female sixth graders: the CReActivity Project. Int J Environ Res Public Health. (2021) 18:1. 10.3390/ijerph18010032 33374568 PMC7793121

[40] Brønd JC Grøntved A Andersen LB Arvidsson D Olesen LG. Simple method for the objective activity type assessment with preschoolers, children and adolescents. Children. (2020) 7:7. 10.3390/children7070072 32630836 PMC7401882

[41] Choi L Liu Z Matthews CE Buchowski MS. Validation of accelerometer wear and nonwear time classification algorithm. Med Sci Sports Exerc. (2011) 43:2. 10.1249/MSS.0b013e3181ed61a3 20581716 PMC3184184

[42] Hänggi JM Phillips LRS Rowlands AV. Validation of the GT3X ActiGraph in children and comparison with the GT1M ActiGraph. J Sci Med Sport. (2013) 16:1. 10.1016/j.jsams.2012.05.012 22749938

[43] Dessing D de Vries SI Hegeman G Verhagen E van Mechelen W Pierik FH. Children's route choice during active transportation to school: difference between shortest and actual route. Int J Behav Nutr Phys Act. (2016) 13:48. 10.1186/s12966-016-0373-y 27072922 PMC4830076

[44] Goodman E Adler NE Kawachi I Frazier AL Huang B Colditz GA. Adolescents' perceptions of social status: development and evaluation of a new indicator. Pediatrics. (2001) 108:2. 10.1542/peds.108.2.e31 11483841

[45] Wen LM Fry D Rissel C Dirkis H Balafas A Merom D. Factors associated with children being driven to school: implications for walk to school programs. Health Educ Res. (2008) 23:2. 10.1093/her/cym043 17884835

[46] Mendoza JA Cowan D Liu Y. Predictors of children's active commuting to school: an observational evaluation in five US communities. J Phys Act Health. (2014) 11:4. 10.1123/jpah.2012-0322 PMC3749259 23575275

[47] Burgueño R González-Cutre D Sevil-Serrano J Herrador-Colmenero M Segura-Díaz JM Medina-Casaubón J. Understanding the motivational processes involved in adolescents' active commuting behaviour: development and validation of the behavioural regulation in active commuting to and from school (BR-ACS) questionnaire. Transp Res Part F. (2019) 62:615–25. 10.1016/j.trf.2019.02.016

[48] Burgueño R González-Cutre D Sevil-Serrano J Herrador-Colmenero M Segura-Díaz JM Medina-Casaubón J. Validation of the basic psychological need satisfaction in active commuting to and from school (BPNS-ACS) scale in Spanish young people. J Transp Health. (2020) 16:100825. 10.1016/j.jth.2020.100825

[49] Heidemann K Hufgard V Sindern E-M Riek S Rudinger G. Das Verkehrsquiz. Evaluationsinstrument zur Erreichung von Standards in der Verkehrs-/Mobilitätserziehung der Sekundarstufe. Mensch und Sicherheit. (2009) M205.

[50] Wartha O Lämmle C Kobel S Wirt T Steinacker JM. Aufbau des Bewegungsmoduls des schulbasierten Gesundheitsförderprogramms “Komm mit in das gesunde Boot”. Dtsch Z Sportmed. (2017) 68:20–6. 10.5960/dzsm.2016.265

[51] Watson A Timperio A Brown H Best K Hesketh KD. Effect of classroom-based physical activity interventions on academic and physical activity outcomes: a systematic review and meta-analysis. Int J Behav Nutr Phys Act. (2017) 14:1. 10.1186/s12966-017-0569-9 28841890 PMC5574081

[52] Morton KL Atkin AJ Corder K Suhrcke M van Sluijs EMF. The school environment and adolescent physical activity and sedentary behaviour: a mixed-studies systematic review. Obes Rev. (2016) 17:2. 10.1111/obr.12352 26680609 PMC4914929

[53] Heath GW Parra DC Sarmiento OL Andersen LB Owen N Goenka S. Evidence-based intervention in physical activity: lessons from around the world. Lancet. (2012) 380:9838. 10.1016/S0140-6736(12)60816-2 22818939 PMC4978123

[54] Standing Conference of the Ministers of Education and Cultural Affairs (KMK). Empfehlung zur Mobilitäts- und Verkehrserziehung in der Schule. (2012). Available online at: https://www.kmk.org/fileadmin/Dateien/veroeffentlichungen_beschluesse/1972/1972_07_07-Mobilitaets-Verkehrserziehung.pdf (accessed Jan 22, 2021).

[55] Yang X Telama R Hirvensalo M Tammelin T Viikari JSA Raitakari OT. Active commuting from youth to adulthood and as a predictor of physical activity in early midlife: the young Finns study. Prev Med. (2014) 59:5–11. 10.1016/j.ypmed.2013.10.019 24201092

[56] Institute of Medicine (IOM). Educating the Student Body: Taking Physical Activity and Physical Education to School. Washington, DC: The National Academies Press (2013). 24851299

[57] Larouche R Mammen G Rowe DA Faulkner G. Effectiveness of active school transport interventions: a systematic review and update. BMC Public Health. (2018) 18:1. 10.1186/s12889-017-5005-1 29390988 PMC5796594

[58] Bjørkelund Børrestad LA Østergaard L Andersen LB Bere E. Associations between active commuting to school and objectively measured physical activity. J Phys Act Health. (2013) 10:6. 10.1123/jpah.10.6.826 23070938

[59] Collard DCM Chinapaw MJM van Mechelen Mechelen W Verhagen EALM. Design of the iPlay study. Systematic development of a physical activity injury prevention programme for primary school children. Sports Med. (2009) 39:11. 10.2165/11317880-000000000-00000 19827858

[60] Anselma M Altenburg TM Emke H van Nassau F Jurg M Ruiter RAC. Co-designing obesity prevention interventions together with children: intervention mapping meets youth-led participatory action research. Int J Behav Nutr Phys Act. (2019) 16:1. 10.1186/s12966-019-0891-5 31831006 PMC6909512

[61] Blümle A Meerpohl JJ Wolff R Antes G. Evidenzbasierte medizin und systematische übersichtsarbeiten. Die Rolle der Cochrane Collaboration. MKG-Chirurg. (2009) 2:86–92. 10.1007/s12285-009-0081-6

[62] Kobel S Wartha O Wirt T Dreyhaupt J Lämmle C Friedemann E-M. Design, implementation, and study protocol of a kindergarten-based health promotion intervention. Biomed Res Int. (2017) 2017:4347675. 10.1155/2017/4347675 28303253 PMC5338306

[63] Lloyd JJ Logan S Greaves CJ Wyatt KM. Evidence, theory and context – using intervention mapping to develop a school-based intervention to prevent obesity in children. Int J Behav Nutr Phys Act. (2011) 8:73. 10.1186/1479-5868-8-73 21752261 PMC3152876

